# A Rare Case of Papillary Thyroid Carcinoma in the Thyroglossal Duct Cyst of a 14-Year-Old Female Patient With Left Thyroid Hemiagenesis

**DOI:** 10.7759/cureus.49712

**Published:** 2023-11-30

**Authors:** Yanko G Yankov, Lyuben Stoev, Simeon Dimanov, Martina Stoeva, Katerina Stanislavova

**Affiliations:** 1 Maxillofacial Surgery, University Hospital St. Marina, Varna, BGR; 2 General and Operative Surgery, Medical University of Varna, Varna, BGR; 3 General and Clinical Pathology, Forensic Medicine and Deontology, Medical University of Varna, Varna, BGR; 4 Oral Surgery, Medical University of Varna, Varna, BGR

**Keywords:** malignant cyst, median neck cyst, thyroid gland, congenital anomalies, cancer in children, neck cancer, head and neck surgery, neck pathology, maxillofacial surgery, pediatric surgery

## Abstract

Congenital abnormalities in the development of the thyroglossal duct are a common pathology in the pediatric population. The exact frequency of hemiagenesis of the thyroid gland is not known because the condition is rarely manifested clinically and is almost always discovered incidentally. Papillary carcinoma of thyroglossal cysts is relatively uncommon, has a good prognosis if promptly detected and treated and occurs mainly in adults. The case we present here is an extremely rare occurrence: a patient with papillary thyroid carcinoma of the thyroglossal duct cyst and thyroglossal duct cyst carcinoma (TDCa). So far, only two such adult patients (women aged 24 and 35) have been described in the world medical literature. The patient we present is a 14-year-old female and is the first described adolescent with papillary carcinoma of the thyroglossal duct cyst and thyroid hemiagenesis (THA).

The disease didn’t have any clinical manifestations, and the patient was brought in by her parents to improve her aesthetic appearance. Neither the physical examination nor the radiological evaluation showed any signs of malignancy. The diagnosis was reached by our team only after the patoanatomical examination. In this patient's case, due to its early diagnosis, the spread of the disease was limited only to the borders of the thyroglossal duct cyst and the absence of regional and distant metastasis. Surgical removal led to a complete cure, without any postoperative data suggestive of residual disease. The functions of the thyroid gland in her case were not affected, despite her left-lobe agenesis, to which there are multiple proofs, namely the normal blood concentration of the examined thyroid markers: free triiodothyronine (FT3), free thyroxine (FT4), thyroglobulin (TG), thyroid stimulating hormone (TSH), anti-TG (thyroid antibody test (TAT)), anti-thyroid peroxidase (TPO) (microsomal antibody test (MAT)), and normal physical and psychological development.

## Introduction

Congenital anomalies of the thyroglossal duct represent one of the most common etiologies for pediatric neck masses, second only to lymphadenopathy [1]. The thyroglossal duct is a transient epithelial-lined midline channel serving as the path of descent of the thyroid primordium from the foramen cecum down to the thyroid cartilage, where definitive thyroid formation occurs [2]. Malignancies arising from thyroglossal duct cysts are very rare in the pediatric population. The clinical behavior of these tumors in the pediatric setting is obscure, and there are no well-defined treatment strategies [3]. In the current case, we describe a 14-year-old female patient with a neck lump and complaints of local discomfort. To our knowledge, this is the first case report in the pediatric age group demonstrating simultaneous occurrence of papillary carcinoma arising in a thyroglossal duct cyst on the background of left THA. 

## Case presentation

A 14-year-old female patient was brought by her parents for an examination at the Clinic of Maxillofacial Surgery, University Hospital St. Marina (Varna, Bulgaria) for active treatment in the spring of 2023. She presented with discomfort and an increase in the size of a lump in the neck region a few months ago, without accompanying symptoms. No medical help was sought, and no diagnostic or treatment procedures were carried out before presenting at our clinic. In the fall of 2016 (at the age of 8), she was hospitalized in the ENT clinic at the same medical facility, where a tonsillectomy was performed on a hypertrophied palatine tonsil. Then, during the physical examination, no deviation from the anatomical norm was found in the neck area, and imaging studies were not conducted. The patient had no accompanying diseases and did not report any family disease history or known allergies to food or medications.

**Figure 1:**
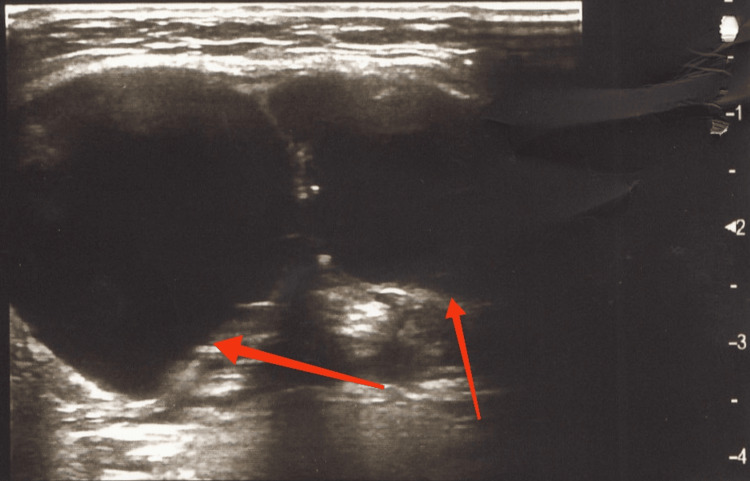
An ultrasound image of the neck in the area of the pathological finding reveals the presence of a multiseptated cyst measuring 48 mm in diameter, containing several smaller cysts filled with fluid

A physical examination by a maxillofacial surgeon revealed the presence of a painless, soft-elastic swelling in the middle of the neck anteriorly, between the lower jaw and the larynx, measuring approximately 5 cm in diameter, with no change in the overlying skin. While swallowing, the mass moved up and forward together with the larynx and hyoid bone. An ultrasound examination of the neck revealed a multisepted cyst 48 mm in diameter containing several smaller cysts, all filled with fluid contents (Figure 1). It was found that the left lobe of the thyroid gland was missing and that the right lobe was compensatory and slightly enlarged. In the area of the missing left lobe, a single cystic lesion measuring 4 mm in diameter with the sonographic characteristics of a simple cyst was visualized. No pathologically enlarged and altered cervical lymph nodes were seen.

**Figure 2:**
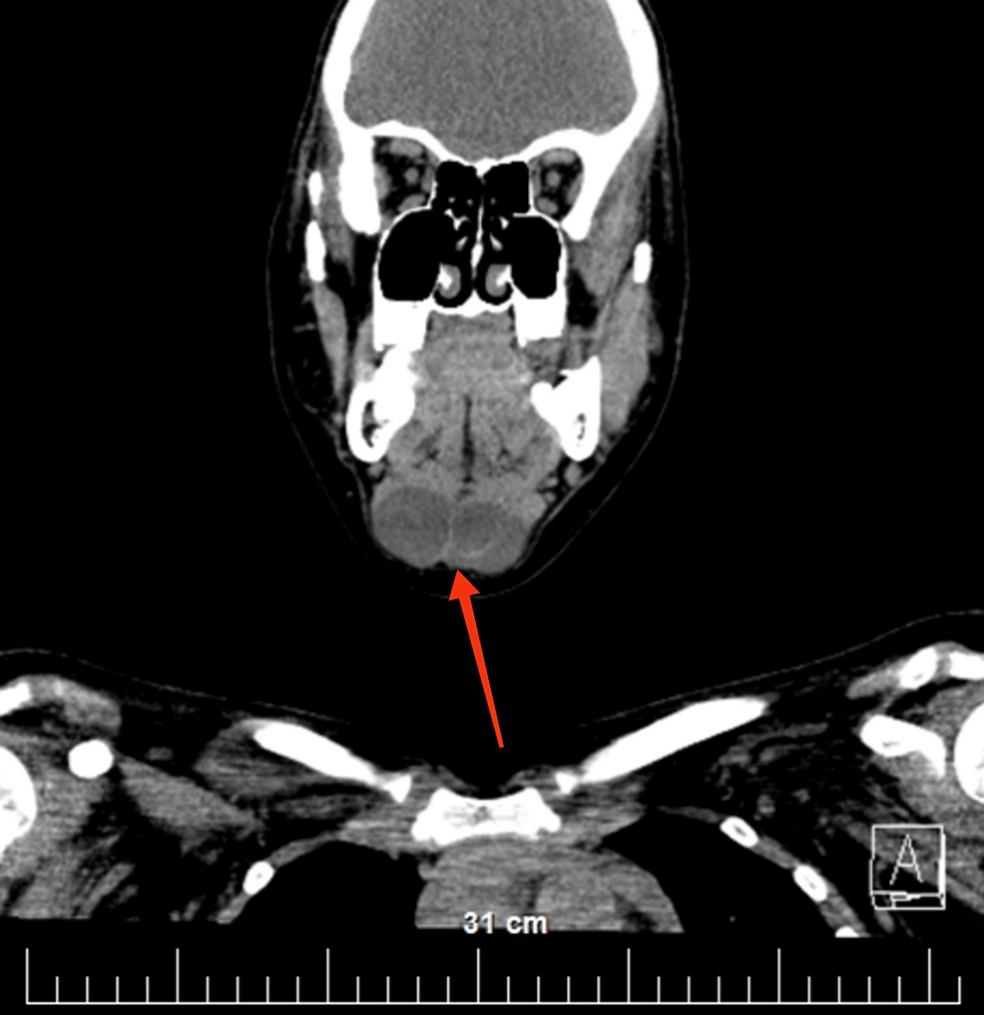
Axial view of the CT (with intravenous contrast) head and neck shows a complex cyst with a three-chambered structure (red arrow)

**Figure 3:**
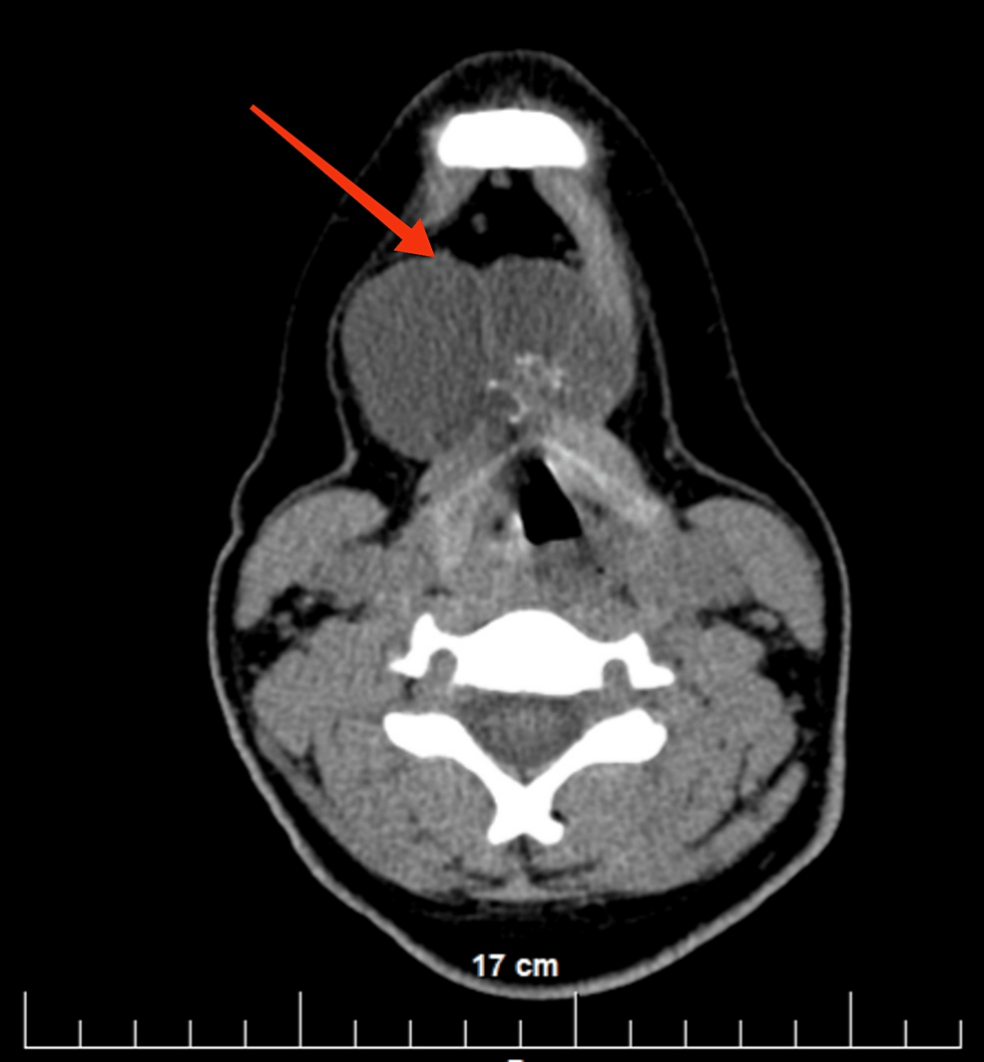
Coronal view of the CT (with intravenous contrast) head and neck shows a complex cyst with a three-chambered structure (red arrow)

The patient was then referred for CT (native and after intravenous contrast) of the head and neck, which confirmed the described complex cyst and showed that its structure was three-chambered (Figures 2-3). After the administration of the venous contrast, no accumulation of the contrast material was observed in it. A blood vessel with small branches is visualized in the central area of the cystic lesion.

**Figure 4:**
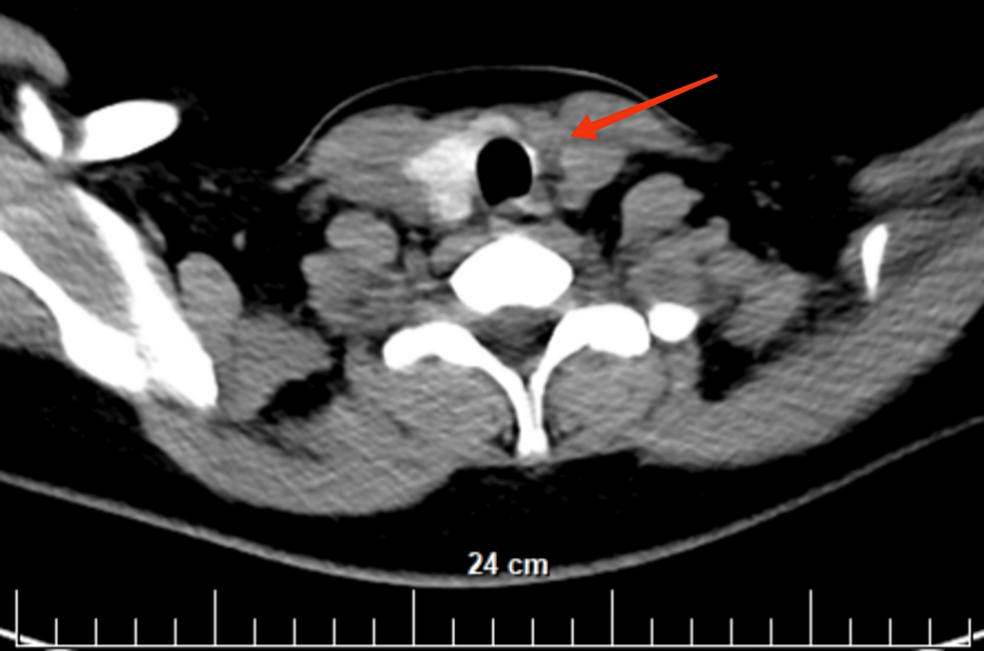
Coronal view of the CT (with intravenous contrast) head and neck shows the absence of the left lobe in the thyroid gland and an enlarged right lobe (red arrow)

The absence of the left lobe of the thyroid gland and an enlarged right lobe were confirmed. No cervical lymphadenomegaly was found (Figure 4).

Based on the information from the anamnesis, physical examination, and imaging studies, the patient was diagnosed with a complex (multiseptated) thyroglossal cyst of the neck and a congenital absence of the left lobe in the thyroid gland. A decision was made for surgical treatment, i.e., the excision of the cyst, following the consent obtained from the patient's parents.

During hospitalization, preoperatively, a second ultrasound of the neck was performed, which confirmed the conclusion from the previous ultrasound and CT. The operative intervention was performed under general anesthesia with orotracheal intubation. After thorough antiseptic treatment of the operative field with Braunol, a horizontal incision was made in the skin, subcutaneous tissue, fascia, and platysma in the upper cervical third, above the existing swelling, and the superficial part of the cyst was visualized. From its lateral surfaces, the infrahyoid muscles were dissected, and they were elevated laterally. It was seen that the formation was firmly connected to the body of the hyoid bone, which necessitated the elevation of the suprahyoid and infrahyoid muscles connected to the median part of the bone, and the body of the latter was removed en bloc with the cystic lesion with an osteotome. The excised material was sent for permanent histological examination to the Clinic of General and Clinical Pathology at our hospital. With a resorbable polyfilament suture, the upper to lower and the right to left suprahyoid and infrahyoid muscles were sutured, but not very tightly. A vacuum Redon drain was placed and activated. The platysma and subcutaneous tissue were sutured with an absorbable polyfilament suture. The skin was sutured with a non-absorbable monofilament suture. A dry, sterile dressing was applied.

**Figure 5:**
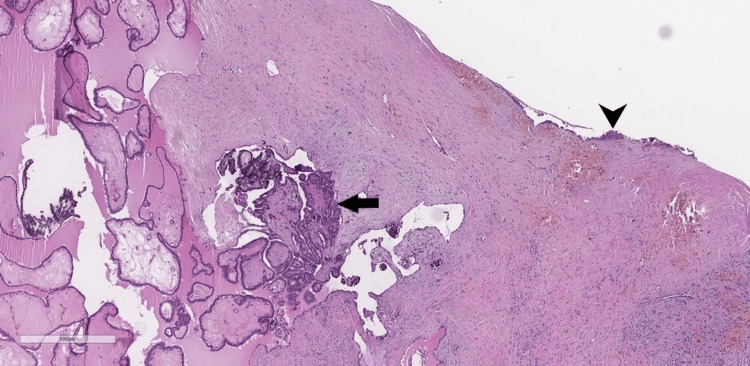
Photomicrograph of the thyroglossal duct cyst, lined by squamous epithelium with subtotal denudation (arrowhead). The cyst wall harbors thyroid-type papillary carcinoma (arrow); H&E x4. H&E: Hematoxylin and eosin

**Figure 6:**
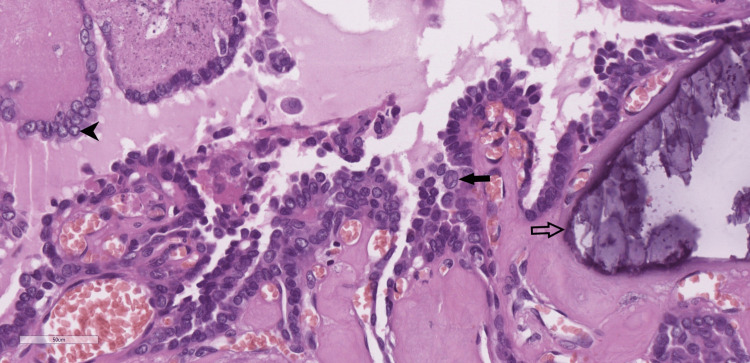
Neoplastic papillae are lined by thyrocytes exhibiting nuclear enlargement and overlapping, chromatin clearing (arrowhead), nuclear grooves, and pseudoinclusions (black arrow). Calcifications are evident on the right (outlined arrow); H&E x40. H&E: Hematoxylin and eosin

The histopathological report revealed papillary carcinoma, arising from ectopic thyroid parenchyma in the wall of the thyroglossal duct cyst (Figures 5-6). There were no findings suggestive of invasion of the hyoid bone and the surrounding musculature.

On the 30th postoperative day, the patient underwent a CT (native and after the intravenous administration of contrast material) of the head, neck, and chest. The imaging excluded the presence of a formation in the area of the previous surgical intervention, confirmed the absence of the left lobe of the thyroid gland, and did not detect cervical lymphadenomegaly. The patient underwent blood tests to determine the plasma concentration of the following hormones and markers: free triiodothyronine (FT3), free thyroxine (FT4), thyroglobulin (TG), thyroid stimulating hormone (TSH), anti-TG (thyroid antibody test (TAT)), anti-thyroid peroxidase (TPO) (microsomal antibody test (MAT)). They were all within normal reference values for her age and sex.

On the 40th postoperative day, a whole-body positron emission tomography (PET)/CT was performed, which ruled out tumor persistence in the neck region and metastases in lymph nodes and other organs. The patient was given the final diagnosis of papillary carcinoma in ectopic thyroid parenchyma in a thyroglossal cyst wall and congenital absence of the left lobe of the thyroid gland. The staging of the tumor process is T1N0M0.

The patient was presented to a pediatric oncology committee, where a decision was made to dispense her for observation. Six months postoperatively, two examinations by a pediatric oncologist and five examinations by a maxillofacial surgeon were performed, and no pathology was found in the neck area. The postoperative cicatrix was normotrophic, and she had no complaints.

## Discussion

Thyroid hemiagenesis is an uncommon congenital disorder of the thyroid gland, characterized by the absence of one lobe. The true prevalence of this condition is well documented because the absence of one thyroid lobe usually manifests clinically but is estimated to be between 0.02% and 0.2% [4,5]. The most typical patterns of THA are either isolated left lobe agenesis or combined THA of the right lobe with concomitant isthmus absence [6]. The frequency of thyroid abnormalities in patients with THА varies with age due to the longer exposure of the hemi-agenetic gland to TSH overstimulation in older patients. This could explain the controversy about the benign character of this anomaly [4].

Thyroid hemiagenesis can be found in association with clinical pathologies such as multinodular goiter, de Quervain thyroiditis, hyperthyroidism, thyroid adenomas, Graves’ disease, or Hashimoto’s thyroiditis [7-10]. Anatomic abnormalities with THA include the absence of an isthmus, the presence of a thyroglossal duct cyst, a sublingual ectopic thyroid, cervical thymic cysts, the absence of the thyroid superior and inferior thyroid vessels, and superior or recurrent laryngeal nerves ipsilateral to the missing lobe, as well as the loss of the parathyroid gland [8]. The L-thyroxine treatment to normalize TSH levels may be beneficial in order to avoid these possible thyroid complications [11].

The thyroglossal duct cyst is a developmental structure participating in the embryogenesis of the thyroid gland. It represents a canal for the descent of the thyroid gland from the ventral aspect of the pharynx. After the migration of the thyroid gland to the thyroid cartilage, the thyroglossal duct cyst goes through atrophic changes and disappears around the 10th week of gestation. Rarely, the duct does not achieve complete regression, and its remnants lead to the formation of thyroglossal duct cysts, usually presenting as a cystic mobile midline anomaly in the anterior neck [12].

As with most cysts, the attenuation of thyroglossal duct cysts is usually between 0 and 20 HU at CT, with a high T2 signal and an intermediate T1 signal on MRI. The fluid within the lesion can be proteinaceous or hemorrhagic, making the lesion higher in attenuation and higher in T1 signal. Most lesions are well circumscribed with a very thin rim of enhancement. Lesions that are infected or hemorrhagic can appear heterogeneous and complex, with profuse surrounding soft-tissue edema. Thyroglossal duct cysts may be distinguished from other cystic lesions when a component closely associated with the hyoid bone is discovered [13].

The histological examination commonly reveals a cyst with either squamous or pseudostratified ciliated columnar epithelium lining; the cyst's wall usually contains ectopic thyroid parenchyma, but this is not an obligatory diagnostic feature [14]. Inflammatory infiltrates are almost invariably present. In some cases, it can be so pronounced that it damages the epithelial lining to the point that it is completely substituted by granulation tissue or fibrosis, significantly perplexing the diagnostic process [15]. The Sistrunk procedure is the gold standard for surgical treatment. It involves the removal of the hyoid body as well as the entire thyroglossal duct tract and a portion of the tongue base to minimize local recurrence [2].

Malignant growth is expected to occur in a small minority (3.2%) of cases with thyroglossal duct cysts [12]. The histopathological result almost invariably reveals thyroid-type papillary carcinoma, while mixed types (papillary and follicular carcinoma), squamous cell carcinoma, and other entities occur far less often [12,16,17]. Tumor-node-metastasis staging of thyroglossal duct cyst carcinoma (TDCa) is currently not advocated by the American Joint Committee of Cancer (AJCC) [18].

In the current case, staging was performed by using extrapolated data from the AJCC 2010 criteria for thyroid gland malignancies, an approach recommended by Thompson et al. [12]. Due to the rarity of the malignant transformation, there are no well-established treatment strategies in cases of unexpectedly encountered neoplasia during the histological examination of the resection specimen. In such situations, the current approach is not to conduct any further surgical treatment if the criteria of Kristensen are fulfilled, i.e., the presence of histologically benign ectopic thyroid parenchyma in the cyst wall, no extension of the tumor through the cyst wall, normal thyroid gland, and no cervical lymph node involvement [19].

Most of the malignancies arising in thyroglossal duct cysts are reported in patients in the 4th decade of life. Although the thyroglossal duct cyst is usually clinically presented in the pediatric age group, malignant change is extremely rare in children [3]. The current case report describes an even rarer constellation of pediatric thyroglossal duct cyst-derived papillary carcinoma arising on the background of THA. To our knowledge, this is the first case reported in the literature in this age group; there are two other cases sharing the same pathology but occurring in older patients: a 24-year-old and a 35-year-old woman [20,21]. In all three described cases, thyroidectomy was avoided due to the fulfillment of the criteria of Kristensen and without any negative clinical consequences.

Papillary carcinoma arising in the thyroglossal duct cyst, when managed with the Sistrunk procedure without thyroidectomy, has an excellent prognosis in the pediatric population. Thompson et al. analyzed 59 patients less than 21 years of age and reported that even patients with soft tissue extension and the presence of lymph node metastasis enjoy favorable outcomes. From the 45 cases with follow-up data, only one patient reportedly died of the disease [3].

## Conclusions

Thyroglossal duct cyst carcinoma represents an extremely rare occurrence in pediatric patients. It commonly behaves as an occult lesion without any substantial clinical or radiological manifestations until it is revealed during the histopathological examination of the resection specimen. In spite of the rarity of these tumors and the lack of definitive consensus for staging and therapy, a relatively conservative approach seems to be optimal regarding the treatment of these lesions.
